# Crystallization of VPS34 with Small Molecule Drug Candidates

**DOI:** 10.1063/4.0001134

**Published:** 2025-10-27

**Authors:** James D Moody, Wisdom Abiodun, Evan Tsubaki

**Affiliations:** Brigham Young University, Provo, Utah, USA

## Abstract

Understanding a protein’s structure is essential for determining its function and for designing drug candidates with high specificity to treat related diseases. We employ X- ray crystallography to determine the structure of target proteins because it gives structural data at the atomic level. This accuracy of detail is critical to design small- molecule drug inhibitors that can effectively modulate protein activity. VPS34 is a lipid kinase that functions in signaling pathways to facilitate cell proliferation, differentiation, and metabolism. The dysregulation of VPS34 is frequently associated with cancer development. As of now, there are no VPS34 inhibitors that have been approved by the FDA, in part due to insufficient specificity of binding which could lead to adverse health effects. We synthesized a small molecule drug candidate, RD-I-53 which specifically inhibits two kinases, JAK1 and VPS34, as determined by a SAR study. This is a notable feat as most kinase inhibitors are promiscuous. We solved the structure of RD-I-53 bound VPS34 (PDB: 9DKP), thus helping us to further optimize the drug candidate.

Some of these derivatives (RD-I-86 (PDB: 9NIN), RD-II-81, RD-I-137, and RD-II-83) have also been structurally characterized bound to VPS34. Through this research, we will contribute to the ongoing efforts in precision medicine and the development of targeted therapeutic interventions.

